# Harm reduction readiness for illicit IV drug use among safety-net primary care practices in the San Fernando Valley

**DOI:** 10.1186/s13690-022-00855-w

**Published:** 2022-04-06

**Authors:** Thomas Kalinoski, Cassandra DeWitt, Hrishikesh Belani

**Affiliations:** 1 Department of Internal Medicine, Olive View – UCLA Medical Center, 14445 Olive View Dr, Sylmar, CA 91342 USA; 2 David Geffen School of Medicine at UCLA, 10833 Le Conte Ave, Los Angeles, CA 90095 USA

**Keywords:** Harm reduction, Injection drug use, HIV, Hepatitis C, Communicable disease

## Abstract

**Background:**

Harm reduction is an accumulation of strategies aimed at preventing adverse health outcomes associated with illicit substance use. Several harm reduction programs and services exist within Los Angeles County (LAC), however their success relies in part on the application of harm reduction principles by local primary care providers serving patients with substance use disorders. This study aims to assess the readiness of patient-centered medical homes in the San Fernando Valley to provide effective harm reduction to patients who use injection drugs and identify barriers to doing so.

**Methods:**

An online survey was distributed to primary care providers and social workers via email at federally qualified health centers and LAC Department of Health Services clinics in the San Fernando Valley between May and June 2019. It consisted of 22 multiple-choice, Likert scale, and short answer questions. The survey assessed knowledge of injection drug use (IDU), familiarity and utilization of harm-reduction and resources, and self-evaluation of attitudes and skills.

**Results:**

There were a total of 41 survey respondents across all clinics. Of respondents, 98% correctly identified heroin as a drug typically injected, and 93% identified Hepatitis C as an infectious risk of IDU. 63% of respondents use harm reduction strategies every few months or less. 34% prescribe buprenorphine routinely, and 76% prescribe pre-exposure prophylaxis to those at risk for Human Immunodeficiency Virus (HIV). 76% are comfortable discussing IDU with their patients, but 59% indicate that they lack the necessary skills, and 42% agree that they lack the time to address it.

**Conclusion:**

Knowledge of IDU was adequate among those surveyed, although overall utilization of harm reduction was infrequent. There is a perceived deficit in skills and time to effectively provide harm reduction to primary care patients in the San Fernando Valley.

## Background

A rise in opioid use disorder (OUD) in the United States has come to national attention in recent years, with the number of opioid overdose deaths being five times higher in 2016 compared with 1999 [1]. This has hit Los Angeles County especially hard, with the prevalence of opioid misuse and abuse higher than the national average [2]. The prevalence of illicit injection drug use (IDU) has risen correspondingly, estimated at up to 1 million people as of 2011. Especially worrisome, IDU carries increased risks for overdose death and the added risks of transmission of hepatitis C and HIV infection, abscesses, and endocarditis [3, 4].

Harm reduction is a set of practical strategies and ideas aimed at reducing these negative consequences associated with drug use described above [5]. Within the San Fernando Valley of Los Angeles County (LAC), there are several harm reduction programs and services available to those who suffer from substance use disorder. These include syringe exchange programs, medication-assisted treatment (MAT), mental health programs, shelters, domestic violence and sexual assault resources, and legal and immigration services [6–8]. The patient centered medical home (PCMH) plays a central role in linking patients with OUD to these services, as the primary care provider (PCP) is often a first point of contact and identification of need.

The goal of our project was to assess the readiness of PCMHs in the San Fernando Valley to provide effective harm reduction to their patients with substance use disorders, and to identify barriers and knowledge gaps. Specifically, we had three main study objectives we wished to accomplish. For the purpose of this study, “health care workers” encompasses healthcare providers – those able to provide healthcare services including MD or DO physicians, nurse practitioners (NP), and physician assistants (PA) – as well as social workers employed in the primary care department of these facilities focused on treating patients with all manner of acute and chronic physical, mental, and social health issues. The first objective was to assess the level of knowledge of health care workers regarding public health risks among injection drug users. The next objective was to assess the level of knowledge and utilization by health care workers regarding harm-reduction and resources available to them. The final objective was to evaluate the attitudes and skills involved with harm reduction.

The geographic area of focus was LAC Service Planning Area 2 (SPA2), which encompasses the majority of the San Fernando Valley. This site was chosen due to proximity to our hospital and the lack of similar assessment completed in this region to our knowledge. We hope that the information gathered may be useful in planning future interventions to improve upon areas of weakness and need in the region.

## Methods

### Study implementation

The study was based on non-probability convenience sampling of health care workers at five safety net adult PCMH settings serving the uninsured and Medicaid populations of SPA2 in LAC out of eight sites originally contacted. These included three federally qualified health centers (FQHCs), as well as the Ambulatory Care Network and Olive View – UCLA Medical Center clinic, which are a part of LA county Department of Health Services (DHS) clinics in the region. A convenience sampling method was chosen due to the ability to get buy-in for participation from local and affiliated sites. Our team contacted the medical directors of these PCMH’s over phone and email to inquire whether their staff were involved in the management of substance use disorders and to request participation of their sites in the study. After written consent obtained, our survey link was forwarded to each medical director for distribution to their staff. The staff included healthcare providers primary care physicians and advance practice providers as well as social workers. The participants were informed (in the introductory e-mail) that the survey was completely voluntary and anonymous, and that they could withdraw from the survey at any time without consequence. The email also included the purpose of the survey and of each set of questions therein. This study was reviewed and approved by the Olive View-UCLA Education & Research Institute Institutional Review Board. Funding for the study software and incentives for survey completion were provided in the form of a grand by the Olive View-UCLA department of medicine.

The survey and introductory email were distributed using the SurveyMonkey® software to the five total participating PCMH sites during the months of May to June 2019. A link to a separate survey was incorporated at the end of the main survey to record participating clinic locations for both demographic information and enrollment into an opportunity drawing. A subset of respondents to the separate survey were randomly selected to receive a twenty-five-dollar gift card as a compensation for time spent participating in the survey. Additionally, the PCMH site with the most responses was awarded a catered lunch. The original protocol was to allow two weeks (5/9/2019 – 5/22/2019) to complete the survey, though due to low participation, a reminder email was sent out on 5/22/2019 and the study extended an additional two weeks until 6/8/2019. Results were recorded and analyzed using the above software as well as Microsoft Excel®. The information was tabulated into basic response rates and organized into bar graphs and word clouds to represent the corresponding values. Data were anonymized, entered into a database and kept in a secure password-protected computer.

### Survey instrument

The survey was originally created by our study team to accomplish the previously outlined objectives using varied question types and subject matter. The survey underwent two phases of validation. The first phase involved feedback from local leaders and experts in harm reduction, as it is defined above, employed within the Los Angeles County Health Department, local non-profit, and academic organizations. In this phase the questionnaire was reviewed for relevance and accuracy to the topic of investigation. The survey was then pilot-tested by a panel of five primary care physicians at Olive View – UCLA Medical Center. Metrics applied for acceptance, modification, or rejection of questions included the following: 1] relevance of each question to our target primary care population 2] question clarity and lack of leading, loaded or double-barreled questions 3] internal consistency. Bias was controlled in the first phase by choosing experts from varied organizations and in the second phase by the selection of faculty across different primary care clinic sites within our institution.

The survey consisted of twenty-two questions, including multiple choice, open-ended short-answer, and 4-point Likert scale responses (strongly disagree, disagree, agree, and strongly agree). The first four questions involved evaluating the demographics of those taking the survey. These demographics included health care worker or practitioner type, years employed, and previous training in harm reduction. The remainder of the survey questions assessed the three broad objectives listed above. The first objective was to assess the knowledge of public health risks of IDU, and was implemented with two multiple choice questions with right or wrong answers based on known statistics. The first of these, a multiple choice question, asked "of the following, which drug typically is used as an injection in the United States?” Answers included synthetic cathinones (“bath salts”), cocaine, phencyclidine, heroin, and methamphetamine. The second of these, also multiple choice, asked “of the following, which infectious risks are associated with injection drug use?” The answers for this question included syphilis, hepatitis A, B, and C, HIV, endocarditis, and abscess. Both of these questions were assigned right or wrong answers based on known published data [9, 10].

The second objective, to assess the knowledge and utilization of harm-reduction resources, was addressed through a combination of nine short-answer questions to record known resources and single-question 4-point Likert scale questions to document the level of utilization. An example question would be “what are the three greatest barriers that you see to successful harm-reduction in your practice?” The last objective, a self-evaluation of attitudes and skills involved with harm reduction, was again applied through 4-point Likert scale to address the self-evaluation and short-answer to get a sense of attitudes and approach to harm reduction with seven total questions. For example, “I have the necessary skills required to treat patients with opiate use disorder (agree or disagree).”

The data was gathered into spreadsheet format and statistics applied. This study mainly utilized descriptive statistics to summarize the data gathered from each question in percentages for the multiple choice questions and modes for the Likert scale questions. As the study was based on convenience sampling with a relatively small sample size, we were not able to apply inferential statistics to our data set.

## Results

A total of 41 total survey respondents participated across all clinics, out of a calculated 104 total health care workers emailed (39%). Among the survey respondents, 16 (39%) were physicians, 9 (22%) were nurse practitioners or physician assistants, and 16 (39%) were employed in social work. Of these health care workers, 23 (55%) have been employed less than 5 years, 8 (20%) employed 5 to 10 years, and 10 (25%) employed over 10 years. The majority of respondents (72%) had never received prior formal training (lecture, structured course or addiction medicine internship or fellowship) in harm reduction strategies.

Of all survey participants, 40 (98%) correctly identified heroin as a drug typically injected. 26 (63%) selected methamphetamine, estimated to be injected by 20% of users. Five (12%) participants selected cocaine of the multiple-choice options, which is more often smoked or snorted but can also be injected [9, 10]. In the question about infectious risks related to injection drug use, 38 (93%) of survey respondents identified Hepatitis C, 40 (98%) identified HIV, and 24 (59%) identified Hepatitis B correctly as infectious risks of injection drug use. Meanwhile, 10 (24%) of respondents selected syphilis and 7 (17%) selected hepatitis A, both of which are indirectly associated risks with IDU [11].

**Fig. 1:**
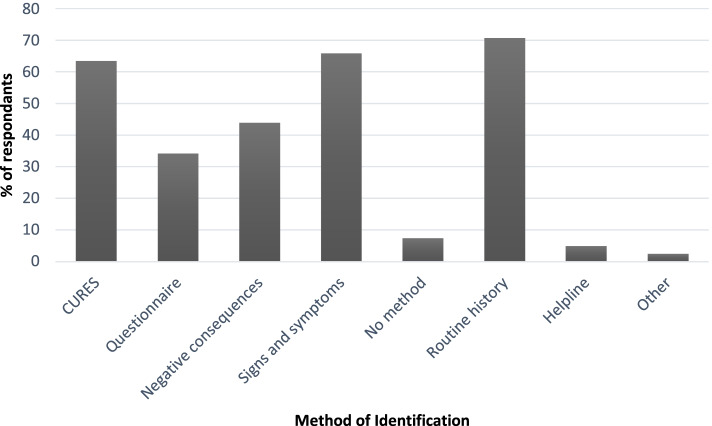
How are you identifying patients at risk for opioid use disorder and IDU?

Twenty-nine (70%) of respondents report identifying patients at-risk for opioid use disorder and injection drug use though routine history-taking in their clinic visits, while 27 (66%) rely on patient-reported signs and symptoms and 26 (63%) use the Controlled Substance Utilization Review and Evaluation System (CURES). Fourteen (34%) of respondents use a dedicated screening questionnaire for opiate use disorder and injection drug use in their clinic (Fig. 1). Furthermore, 41% (17) of all respondents routinely ask their patients precribed opiates about injection drug use, though 76% (31) of respondents reported that they are comfortable discussing IDU with their patients.

Sixty three percent of survey respondents (26) report using harm reduction strategies every few months or less. Seventy-six percent (21) of providers (MD, DO, PA, NP) prescribe naloxone to eligible patients in their practice and 96% (24) routinely screen for HIV and hepatitis C for their patients who inject drugs. Seventy-six percent of providers report routinely prescribing pre-exposure prophylaxis (PreP) for HIV to patients at risk. Of providers who do not prescribe PreP a variety of explanations were given including knowledge gap, time and resource concerns, and that they had not previously considered this as an option for patients who inject drugs. Thirty-two percent (8) of providers have been approved for a waiver to practice MAT with buprenorphine (6 MD or DO and 2 nurse practitioners), though only 6 (75%) of these are currently using it to prescribe buprenorphine routinely. Of those respondents who were eligible but did not have an X-waiver at the time of the survey, 41% (7) were interested in completing training to obtain one. In the free text explanations of the reason why they are not interested almost all respondents listed they were not interested or did not have the time to take on this prescribing role. One anonymous quote described it in this way *“It’s hard already to sit down and listen to patients with all the preventive care and new issues/urgent issues that have to be dealt with in one visit. A visit that is not supported by any ancillary staff. a "good" physician who stays after hours every day, or runs late every clinic is a burnt-out physician*.” Others suggested the solution to this issue is using social workers and substance use or addiction counselors. Other frequently-reported barriers for harm reduction delivery for injection drug use include resource and referral limitations, knowledge/training of providers, patients not ready or interested in harm reduction (Table 1).

**Table 1:** Answers to the Question "What are the 3 greatest barriers to harm reduction?"

What are the 3 greatest barriers to harm reduction?	Number of responses
Resource and referral limitations	17
Time	14
Patient not ready or interested	13
Knowledge/training of providers	11
Other medical or mental health diagnosis	8
Housing	7
Lack of addiction counselors, social work, or multidisciplinary approach	6
Lack of peer or family support	5
Patient follow through	5
Comfort of providers	3
Transportation	3
Patient social situation	3
Language/cultural barriers	2
Problems with screening for substance use	2
Stigma	2
Time delay for resources/referral	2
Difficulty building rapport	1
Low health literacy	1
Chronic pain management alternatives	1

**Fig. 2:**
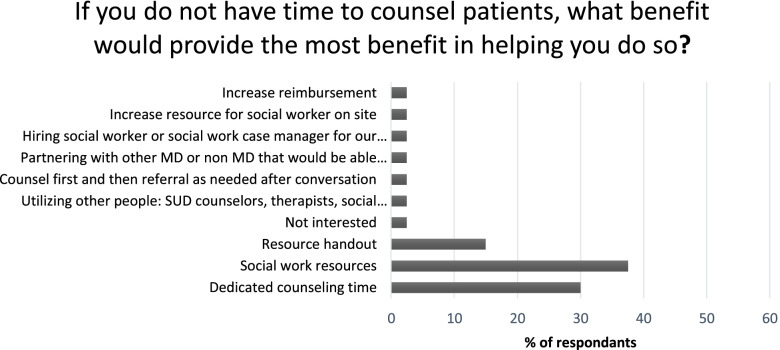
If you do not have time to counsel patients, what intervention would provide the most benefit in helping you do so?

Through the 4-point Likert scale questions, respondents conveyed various levels of preparedness and concerns regarding counseling on harm reduction resources of patients who use injection drugs. The most frequent response to the statement “I am prepared to provide resources for harm reduction to my patients who inject drugs” was “agree” (18) followed by “disagree” (11). Furthermore, 17 respondents disagree with the statement “I have the necessary skills required to treat patients with opioid use disorder”, the most of any respondents. The most frequent response to the statement “I have enough time to counsel patients on harm reduction services” was a “disagree” at 18 responses. Respondents most commonly reported social work resources as a beneficial intervention in time limited settings (Fig. 2). When asked what additional information staff would find helpful, the most common responses included additional trainings, staff with specialization in harm reduction, and easily accessible and up to date resource lists (Table 2).

**Table 2:** Answers to the Question "What are the top 3 clinical interventions most helpful/feasible for you to deliver in your practice to reduce injection drug use-related harm in your patients?"

What are the top 3 clinical interventions most helpful/feasible for you to deliver in your practice to reduce injection drug use-related harm in your patients?	Number of Responses
Motivational interviewing and counseling	29
Referral to treatment center or 12 step program	24
Housing assistance	7
Referral to Needle and syringe exchange program	11
Opiate agonist therapy (buprenorphine, suboxone, methadone)	15
Opiate antagonist therapy (naltrexone)	8
Discussing safe injection practices and providing safe injection kits (sterile water, cottons, cookers, alcohol swabs etc.)	5
Discussing overdose prevention	12
Prescribing Naloxone * *One person selected Other and typed Naloxone so this was added to total	11

## Discussion

Respondents believe that they lack adequate skills to treat patients with opiate use disorder, and reported a need for social work in their practice to address this. Social workers, on the other hand, reported being prepared to provide resources to those who inject drugs. There were also significant challenges described in the limited time for patient encounters and providers indicated resistance to adopting this role and preference to use social workers in time-limited settings.

Of respondents, there seems to be adequate general knowledge of practice-relevant information on injection drug use. This is reflected in the responses to questions regarding knowledge of the personal and public health risks, common injection drugs of abuse, and methods of identifying those at risk. Providers also feel overall prepared to provide harm reduction services to their patients who inject drugs and comfortable discussing injection drug use with their patients. Despite this, there is a limited overall use of harm reduction resources in practice.

Common barriers to effective harm reduction practice were identified as a lack of specific knowledge about harm reduction methods, lack of skills and time to implement them, paucity of ancillary social work staff available in the PCMH and perceived patient unwillingness to engage in interventions. We also identified inconsistent and irregular screening for substance use among our survey respondents.

Many respondents reported not having had any previous formal training in harm reduction. This may have had an effect on the reported lack of knowledge and skills for harm reduction detailed above. The perceived need for harm-reduction specific training and information about local harm reduction resources was a common theme among the different PCMHs in the study. We suggest training implemented by recognized champions in the field who can model desired behaviors to allow providers in the San Fernando Valley to overcome the perceived lack of knowledge and gain the necessary clinical skills for harm reduction practice. PCMHs may consider training modules for harm reduction available online or in-person, and providing lists of harm reduction resources available locally at each clinic and in the surrounding community of the San Fernando Valley. Creating area-specific resource lists would create behavioral support to decrease barriers to referral to harm reduction resource.

One foreseeable barrier to the implementation of harm reduction training programs and increasing the availability of MAT is that both are relatively novel ideas to many health care facilities. The organizations that sponsor and govern these practices may not be recognized or even known to all that could potentially use them. This barrier would be compounded by those facilities that already have issues with funding and availability of resources.

Lack of time to counsel patient on harm reduction strategies with patients may be addressed by scheduling sequential, dedicated appointments, both with the providers and social work staff, to address substance use once identified by routine screening. This would, however, require an increase in co-located social work staff – another gap identified by most respondents. Environmental restructuring in this regard can influence provider behavior by changing the physical or social context in which they work. At a policy level there is a compelling interest for publicly funded insurance, Medicaid and Medicare, to provide reimbursement for harm reduction counseling, paying for additional time for these clinic encounters.

Other gaps identified in our study include low implementation of MAT and relatively low interest in X-waiver training and obtainment. While prescription of naloxone for eligible patients on chronic opioid medication was high in this study, routine screening and assessment of injection drug use behavior among patients was low. Additionally, there is a significant proportion of providers who do not currently offer at-risk patients Pre-Exposure Prophylaxis (PrEP), attributed to a perceived lack of knowledge and training. To promote this practice, we suggest a guideline be introduced, which would provide delineated expectations of care that can be distributed through protocols across different providers and clinics.

There were a few limitations of this study. The main limitation is the low percentage of respondents overall, as well as relatively few total clinics sampled within SPA2. This was likely due to the time constraints with the relatively large size of the questionnaire. Another limitation is the non-probability convenience sampling we used, which was inherent to a study of this type.

## Conclusion

The purpose of this study was to assess the readiness of health workers in the San Fernando Valley to provide effective harm reduction to their patients. We were able to identify multiple barriers to effective harm reduction and implementation, and areas of weakness in knowledge and skills. This information will be useful for planning future targeted interventions to improve access to harm reduction services and hopefully clinical outcomes for patients. This survey also highlighted challenges in primary care due to limited time in appointments and social work support which could be remedied through health care policy changes.

## Data Availability

All datasets generated and analyzed during this study are available from the corresponding author upon reasonable request.

## Abbreviations

LAC: Los Angeles County
IDU: Injection Drug Use
HIV: Human Immunodeficiency Virus
OUD: Opioid Use Disorder
MAT: Medication-Assisted Treatment
PCMH: Patient Centered Medical Home
PCP: Primary Care Provider
SPA2: Service Planning Area 2
FQHCs: Federally Qualified Health Centers
DHS: Department of Health Services
CURES: Controlled Substance Utilization Review and Evaluation System
PreP: Pre-Exposure Prophylaxis
